# Dispensing of antibiotics without prescription in the metropolitan area of Athens, Greece, in 2021—Can new legislation change old habits?

**DOI:** 10.1017/ash.2022.357

**Published:** 2023-03-02

**Authors:** Ioannis Kopsidas, Lydia Kokkinidou, Dioni Pinelopi Petsiou, Eleni Kourkouni, Christos Triantafyllou, Grammatiki-Christina Tsopela, Theoklis Zaoutis

**Affiliations:** 1 Second Department of Pediatrics, National and Kapodisitrian University, Athens, Greece; 2 Center for Clinical Epidemiology and Outcomes Research, Athens, Greece

## Abstract

**Objective:**

To assess the effect of new legislation on the dispensing of antimicrobials without prescription from pharmacies in Greece.

**Design:**

In-person survey.

**Setting:**

The study included 110 pharmacies in the greater Athens Metropolitan area.

**Methods:**

Volunteer collaborators visited 110 pharmacies in the greater Athens Metropolitan area in December 2021 and January 2022. They asked for either ciprofloxacin or amoxicillin-clavulanate acid (6:5 ratio) without providing a prescription, without simulating symptoms, and without offering justification or insisting. Fluoroquinolones have additional dispensing restrictions in Greece. Results were compared to a 2008 study. In 2020, legislation allowed the dispensing of antibiotics from pharmacies only with an electronic prescription, overriding the 1973 forbidding the dispensing of all medications without prescriptions.

**Results:**

All pharmacists refused to dispense ciprofloxacin without a prescription. Only 1 pharmacy dispensed amoxicillin-clavulanate without a prescription. Compared to the 2008 study, dispensing of amoxicillin-clavulanate without a prescription dropped from 100% in 2008 to 1% in 2021 and dispensing ciprofloxacin without a prescription dropped from 53% in 2008 to 0% in 2021.

**Conclusions:**

A new and enforced law that requires electronic prescribing led to a dramatic reduction of antibiotic dispensing without prescription compared to 12 years ago. Similar initiatives could help solve the problem of antibiotic consumption and resistance in Greece and elsewhere.

Antibiotic resistance is still a major global public health problem due to its impact on morbidity, mortality, and healthcare costs. Despite warnings from international organizations,^1–3^ the trend toward antibiotic consumption is continuing to increase in some countries, especially in low- and middle-income countries.^4^ Assuming no policy changes, global antibiotic consumption in 2030 is forecast to be 200% of the 2015 estimate.^4^

Although many factors can explain these increases in antibiotic use and resistance, the dispensing of antibiotics without prescription in community pharmacies is still one of the most important factors.^5,6^ It is quite common, especially in low- and middle-income countries,^7^ and often means self-diagnosis, which is an additional problem. This self-medication is associated with an inadequate use of antibiotics, inadequate dosages, and duration of treatments that increase the risk of resistant bacteria selection.^8^

In Greece, both the use of antibiotics and resistance rates are high. Since 1973, national legislation exists that forbids the dispensing of any medication, including antibiotics, ‘over the counter’ without a prescription (ND 96/1973–FEK 172/A/8-8-1973).^9^ In a 2008 study by Plachouras et al,^10^ which aimed at quantifying dispensing of antimicrobials without prescription in Athens, Greece, researchers procured amoxicillin-clavulanate acid at 100% of the times they asked for it, whereas ciprofloxacin, which required additional special prescription (“justified prescription of administration of newer quinolones”) was dispensed by 53% of pharmacies without a prescription. In 2020, the Greek government readdressed the matter with a new law (4675/2020–FEK54/A/11.03.2020)^11^ that made dispensing antibiotics possible only with an electronic prescription and put an end to handwritten prescriptions, without changing the sanctions imposed. “Justified quinolone prescriptions” were also made electronic.^12^

In this study, we sought to determine whether the dispensing of antimicrobials without prescription from pharmacies in Greece has been limited in the 13 years since the earlier study and after the new legislation was passed.

## Methods

### Study settings

Volunteer collaborators visited 110 pharmacies in the greater Athens metropolitan area in December 2021 and January 2022: 2 female medical students, a male nurse, a retired woman, and a male pediatrician. The power analysis for 110 pharmacies was estimated at 86% to detect differences of 20% in changes of antibiotic dispensing between our 2021 study and the 2008 study. Athens had been divided administratively into 5 regions with their respective populations according to the 2011 census (Table 1).^13^ In 2021, the greater Athens area had 3,200 pharmacies according to the Pharmaceutical Society of Athens (PSA, www.fsa.gr). The number of pharmacies visited in each area were chosen to reflect the respective populations. For General Data Protection Regulation (GDPR) reasons, the PSA declined to provide a list of all pharmacies. Thus, each collaborator was assigned an administrative area, and the pharmacies visited in each area were chosen randomly by the collaborator.

**Table 1.** Dispensing of Antibiotics Without Prescription in Pharmacies by Region and Agent in Athens, Greece, December 2021–January 2022 Versus April–May 2008

			2021 (N=110 Pharmacies)		2008^10^ (N=174 Pharmacies)	
Area	Population, No. (%)	No. of Pharmacies Visited in 2021	Pharmacies Dispensing Ciprofloxacin, No. (%)	Pharmacies Dispensing Amox-Clav, No. (%)	% Pharmacies Dispensing Ciprofloxacin	% Pharmacies Dispensing Amox-Clav
City Center	1,029,520 (32.7)	36	0 (0)	0 (0)	46	100
Northern suburbs	592,490 (18.8)	21	0 (0)	0 (0)	50	100
Western suburbs	489,675 (15.6)	17	0 (0)	1 (12.5)	54	100
Southern suburbs	529,826 (16.9)	18	0 (0)	0 (0)	61	100
Eastern suburbs	502,348 (16.0)	18	0 (0)	0 (0)	Not visited	Not visited
Total	3,143,859 (100.0)	110	0 (0)	1 (1)	53	100

Note. Amox-clav, amoxicillin-clavulanate acid.

Antibiotics in Greece are sold in branded packages and not in generic drug containers. Any other medication (antihypertensives, statins, contraceptives, etc) can be purchased without prescription by asking for a package, even though the 1973 law is still effective, regardless of whether the person providing the drug is a pharmacist or another employee. Most of the time, it is unknown whether the person serving customers is a pharmacist; a pharmacist is not always present. It is common practice for pharmacies to stock all kinds of drugs, amoxicillin-clavunate and ciprofloxacin included. All pharmacies are privately owned small enterprises; only pharmacists are allowed to own pharmacies.

### Study procedure

The voluntary collaborators asked for either ciprofloxacin or amoxicillin-clavulanate acid (6:5 ratio) without providing a prescription or any justification. Amoxicillin-clavulanate acid was selected because it is the most frequently used antibiotic, and ciprofloxacin was chosen because an additional prescription is required. The collaborators were instructed to neither insist, nor pretend any illness or symptom, nor to influence the decision. There was no way of distinguishing between pharmacy employees and pharmacists, and attempting to assess this factor could have compromised the study. The antibiotic was or was not purchased, and the number of pharmacies that dispensed was recorded, along with the reason (if given) for refusing sale and any additional comments made by the pharmacist. In this way, we attempted to duplicate the methodology of the 2008 study. Ethical approval was received before the initiation of the data collection from the bioethics committee of the National and Kapodistrian University of Athens (no. 580/19.11.2021).

### Statistical analysis

Results are presented with absolute and relative frequencies (%). No statistical test or *P* value was provided to compare the 2 dispensing periods (2021 vs 2008) due to very low number of cases of dispensing in 2021. Stratified results are also presented for each geographic area.

#### Data collection

No data were collected that could lead to the identification of the pharmacies. Volunteers destroyed receipts when antibiotics were provided. Only the area of the pharmacy was recorded (northern, eastern, southern, eastern suburbs or the city center), the antibiotic requested, and whether it was given or not.

## Results

All pharmacists refused to dispense ciprofloxacin. Only 1 pharmacy dispensed amoxicillin-clavulanate. Compared to an equivalent study performed in 2008, dispensing of amoxicillin-clavulanate dropped from 100% in 2008 to 1% and ciprofloxacin dropped from 53% in 2008 to 0%. Dispensing by region and by agent in 2008 and 2021 is shown in Table 1. The reason the pharmacists gave was the new legislation.

## Discussion

Our study documented a dramatic change in the dispensing of antibiotics without a doctor’s prescription in the community. In comparison with a prior study in 2008, we only managed to procure 1 box of amoxicillin-clavunate and no ciprofloxacin, compared with 100% and 53% of the times requested, respectively, in 2008.

Self-medication with antibiotics is a worldwide problem that is adding to the overconsumption of antibiotics and antimicrobial resistance. Although regions of the world vary regarding the extent to which one can procure antibiotics without prescription in community pharmacies, a systematic review has estimated that ∼78% of antibiotic requests are successful.^14^ This problem has been described in Greece,^10^ Spain,^15,16^ Pakistan,^17^ China,^18^ sub-Saharan African countries,^19^ and elsewhere.^7^

Laws might exist, but they may not work and may not be enforced. In Spain in 2008, researchers managed to acquire antibiotics without prescription from almost 80% of pharmacies when simulating a urinary tract infection, but they received antibiotics in fewer pharmacies for simulating sore throat or bronchitis.^15^ The problem in Spain remained 10 years later.^16^ Some similar studies have used simulation of clinical symptoms as a way to provoke the dispensing of antibiotics without prescription.^15,20,21^ In Greece, in 2008, the problem was so profound that even without a simulation of a clinical condition or offering any other information to the pharmacists, they were successful in acquiring antibiotics every single time when they asked for amoxicillin-clavunate and almost half of the times they asked for ciprofloxacin.^10^ In another study in Greece a few years earlier, 74.6% of adults had self-reported to have used non-prescribed antibiotics and 22.7% of parents had administered to their children antibiotics without having a prescription.^22^

### What seems to work

Some countries have successfully reduced antibiotic consumption without prescription with the implementation of restrictions. Mexico introduced legislation that required pharmacies to retain and register antibiotic prescriptions, and Brazil required pharmacies to keep copies of the prescriptions.^23^ In Saudi Arabia, even though legislation existed, it was only enforced in March 2018^24^ by the Ministry of Health, which threatened to fine violators and have their licenses revoked. In Chile, enforcement of existing laws decreased antibiotic sales by 43%.^25^ Training pharmacists, but also the public, can aid in tackling the problem of self-medication with antibiotics.^19^ Television and other media can be used to reach a great number of people and educate them on the pitfalls of self-medication with antibiotics.^7^

In Greece, relevant legislation existed since 1973. We are not certain why the 2020 legislation was successful and the previous law was not. One possibility is that electronic prescribing, which started in 2011, was helpful in enforcing the new law. The need for the new legislation obviously indicates that the old law was not effective, and European Centre for Disease Prevention and Control (ECDC) data on community antibiotic consumption seem to support this.^26^ When the new law passed, the country’s pharmacists were warned by their national and local associations to conform to the new law because inspections would be imminent and should be expected. This warning showed the government’s intention to enforce the new law.

## Limitations

Our study was conducted in Athens, a metropolitan area; thus, results might not be generalizable for all of Greece. Personal relationships in rural areas could allow for more relaxed dispensing, but the pharmacist would need to provide an electronic prescription, perhaps with the aid of a complicit local doctor. Our study only assessed the dispensing without a prescription; we were unable to measure whether there was an increase in antibiotic prescribing compared to previous years as a result of the enforcement of new legislation. Not having a prescription and not pretending illness or symptoms could have hindered the success in procuring antibiotics without prescription. Not knowing whether the person refusing the antibiotics was a pharmacist is another limitation. The 2008 study we used for comparison had more or less (if not exactly) the same limitations. No published data on antibiotic dispensing without prescription prior to the new legislation are available; thus, no real baseline measurement exists. However, the ECDC data on community antibiotic consumption, although not comparable directly, show stability if not increases 2015 and 2019.^26^ In 2009, the consumption rate was 34.6 defined daily doses per 1,000 inhabitants per day (DDD), and this rate was >30 DDD from 2015 throughout to 2019. Consumption rates <30 DDD were only documented between 2012 and 2014, following the 2011 implementation of electronic prescribing in Greece. However, a cause-and-effect relationship cannot be assumed.

In Greece, a country plagued by high antimicrobial resistance rates and consumption of antimicrobials, a new law requiring electronic prescribing led to the dramatic reduction in antibiotic dispensing without prescription compared to 12 years earlier. Further studies in rural areas, with or without scripts, and comparison of administrative data for prescription patterns over the last decade could be helpful in acquiring a deeper understanding of this change. Similar initiatives could aid to battle Greece’s problem of antibiotic consumption and resistance.
